# Exposure levels of ELF magnetic fields in the residential areas of Mangaung Metropolitan Municipality

**DOI:** 10.1007/s10661-018-6916-8

**Published:** 2018-08-23

**Authors:** Phoka Rathebe, Carien Weyers, France Raphela

**Affiliations:** 1 Department of Environmental Health, University of Johannesburg, Doornfontein Campus, Johannesburg, 2028, South Africa; 2 Department of Life Sciences, Central University of Technology, Bloemfontein, Free State South Africa; 3 Department of Clinical Sciences, Central University of Technology, Bloemfontein, Free State South Africa

**Keywords:** Residential exposure, Distribution substations, Extremely low frequency, Electromagnetic fields

## Abstract

The aim of this study was to evaluate the exposure levels of ELF magnetic fields in the residential areas of Mangaung metropolitan municipality. Fifteen residential sites were randomly selected in Bloemfontein, nine in Botshabelo and six in Thaba Nchu areas of Mangaung. Measurements were collected at the distances of 3 m, 6 m and 9 m outside electrical substations, near every corner, using a Trifield meter model XE 100. Measurements were also collected from four different corners inside substations, near barrier screening and were referred to as a distance of 0 m (reference point). The results indicated a non-significant difference among 15 residential areas; BRE1 to BRE15 and six areas; TNRE1 to TRNE6. The exposure levels were significantly high in one residential area BORE1 (0.55 μT) as compared to other residential sites in Botshabelo (*p* < 0.001). The results obtained from the measurements also show a significant difference between the residential areas BORE4 and BORE8 (*p* < 0.01) as well as BORE4 and BORE9 (*p* < 0.006). The four distance interims also demonstrated a highly significant difference (*p* < 0.0001) when compared to one another. The *t* test showed a statistically significant difference for exposure levels recorded at 3 m, 6 m and 9 m in comparison to 0 m (*p* < 0.01). The exposure levels recorded at 3 m were also significantly different to those recorded at 6 m (*p* < 0.05) and 9 m (*p* < 0.01). The exposure levels measured at all distances are below the ICNIRP guidelines and the fields decrease rapidly with an increased distance from the source.

## Background

Distribution substations are an integral part of the electrical power supply network. In every community, distribution substations enable the common use of low-to-high-voltage electricity and subsequently generate electromagnetic fields (EMFs) (WHO 2007). The size of distribution substations differs significantly; it depends on the type of property it serves. Substations, and power lines, are regarded as the imperative source of emission of EMFs (WHO 2007). EMFs with a frequency range of 0 to 300 Hz are considered extremely low frequencies (ELFs) (Tworoger et al. 2004), and they are also emitted by the substations. The distribution substations in South Africa, like in most European countries (Greiller et al. 2014), are operated at a frequency of 50 Hz, so public exposure to ELF magnetic fields must be taken into cognizance (WHO 2007). Substations are not the only source of public exposure to ELF magnetic fields in the residential areas, the other sources of concern include household electrical appliances, transmission power lines, the wiring of buildings and electric transportation systems (Barsam et al. 2012). Electrical substations are usually found in the residential areas and people living in close proximity to them are exposed to high levels of ELF magnetic fields (Margallo 2009). Public concerns have raised about the possible health effects of EMF (Tworoger et al. 2004). According to Suhnel and Berg (2003), it is necessary to conduct exposure assessments to determine health effects of ELF magnetic fields among the public.

In South Africa, there is an insufficient data on the residential exposure to ELF magnetic field levels from distribution substations. However, there is existing studies around the development of health effects from exposure to ELF magnetic fields. Recent studies include, but not limited to, assessment of potential health impacts from ELF magnetic fields in the residential areas of Europe, occupational exposure to ELF magnetic fields and natural killer activities in peripheral blood lymphocytes and childhood leukaemia not linked with ELF magnetic fields (Greiller et al. 2014; Gobba et al. 2009; Leitgeb 2014). Large-scale assessments of public exposure to ELF magnetic fields have been conducted not only in Western Europe, but also in the USA, New Zealand, Japan and also in Central Europe, where exposures were measured outside and inside high-rise, multi-family and family houses including time variations (Jirik et al. 2011). According to Schuz and Ahlbom (2008), ELF magnetic fields generated from the distribution and supply of households have drawn an attention due to their presence in the environment. Electrical supply infrastructure such as overhead power lines, electrical substations and domestic appliances are prime contributors of residential exposure to ELF magnetic fields (Vulevic and Osmokrovic 2011; Huss et al. 2013; Leitgeb et al. 2008). In European countries, the ELF magnetic fields exposure assessment is performed to selected environments and certain population (Durrenberger 2012). The aim of this study was to evaluate residential exposure to ELF magnetic fields in the three prominent areas of Mangaung metropolitan municipality. The focus on many studies around ELF magnetic fields have been conducted to evaluate the development of leukaemia in children (Mild et al. 2005) and other long-term health effects but evidence is still non-conclusive (Feytching et al. 2005). Different exposure assessments that have been performed, majority of them were not coordinated, except for studies focusing on residential exposure from substations (Transexpo 2010). As ELF magnetic fields are considered possible carcinogenic “Group 2B” (IARC 2002a, b), this study intends to create public alertness by providing data around ELF magnetic fields emitted by distribution substations in the residential areas of Mangaung metropolitan region.

## Methodology

### Study design

**Fig. 1:**
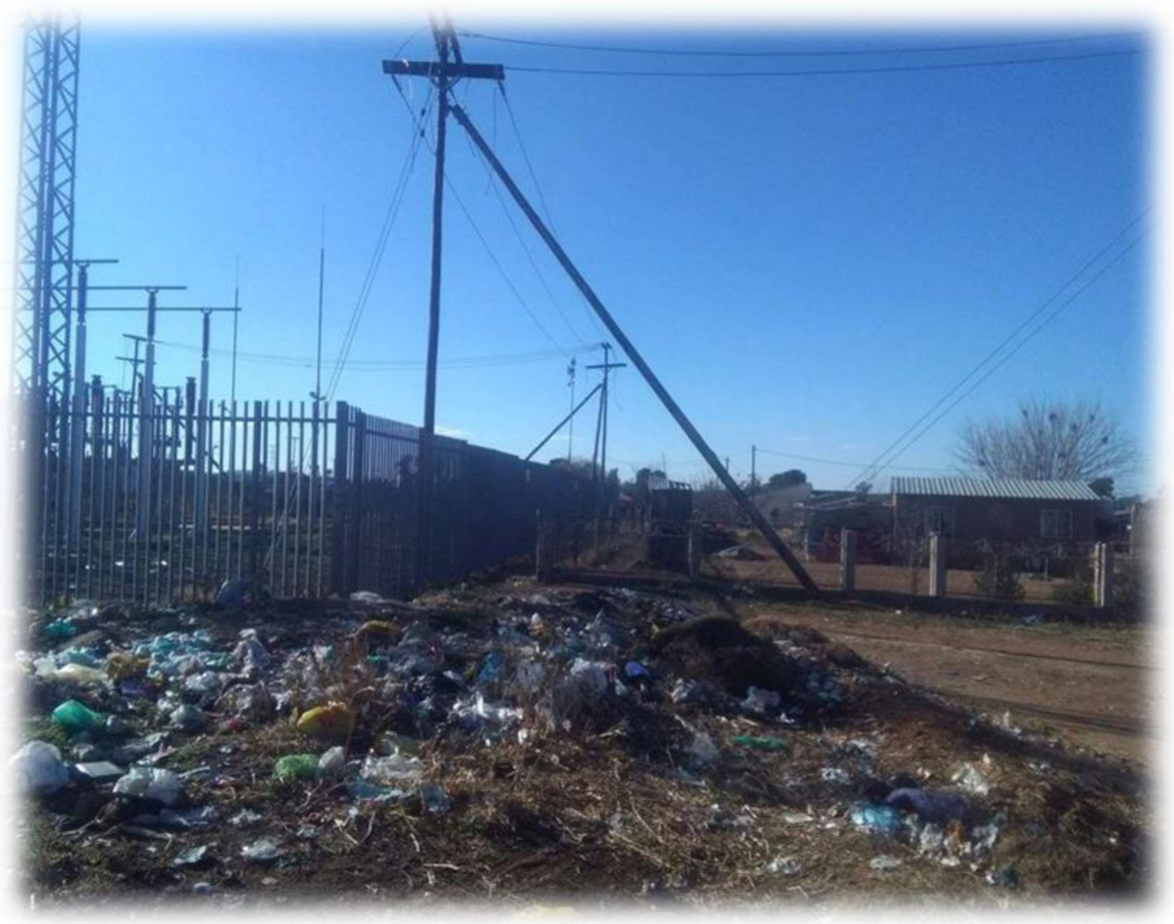
Household in close proximity to BS1

**Fig. 2:**
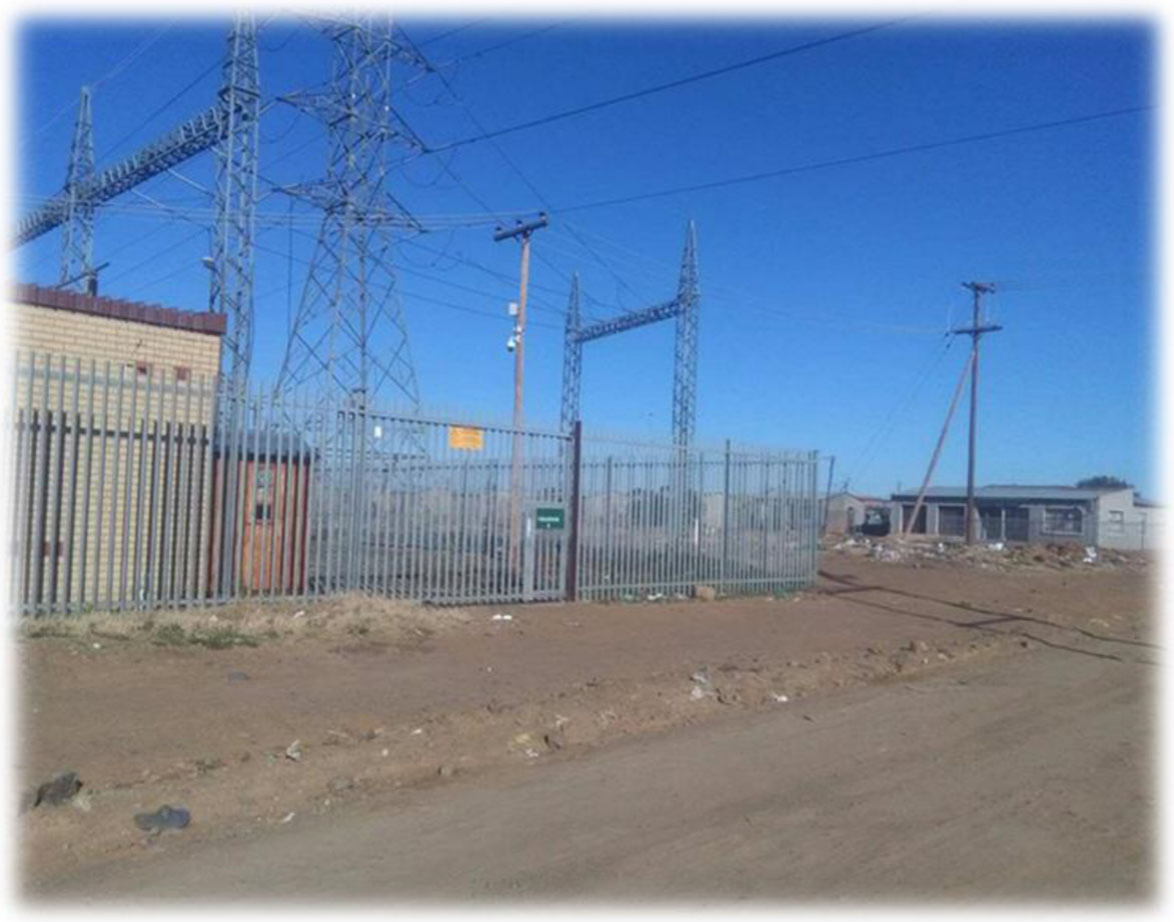
BOS1 nearby residential properties

**Fig. 3:**
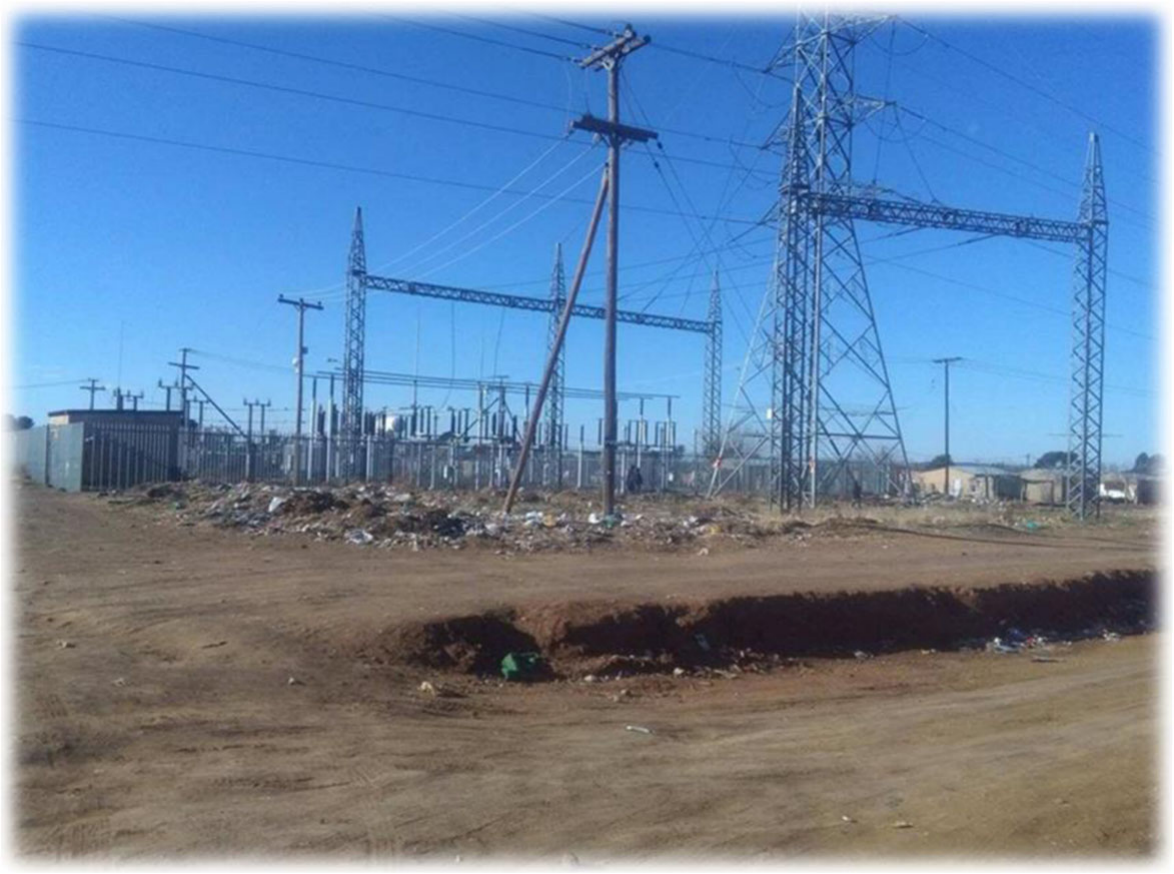
Side view of BS1

**Fig. 4:**
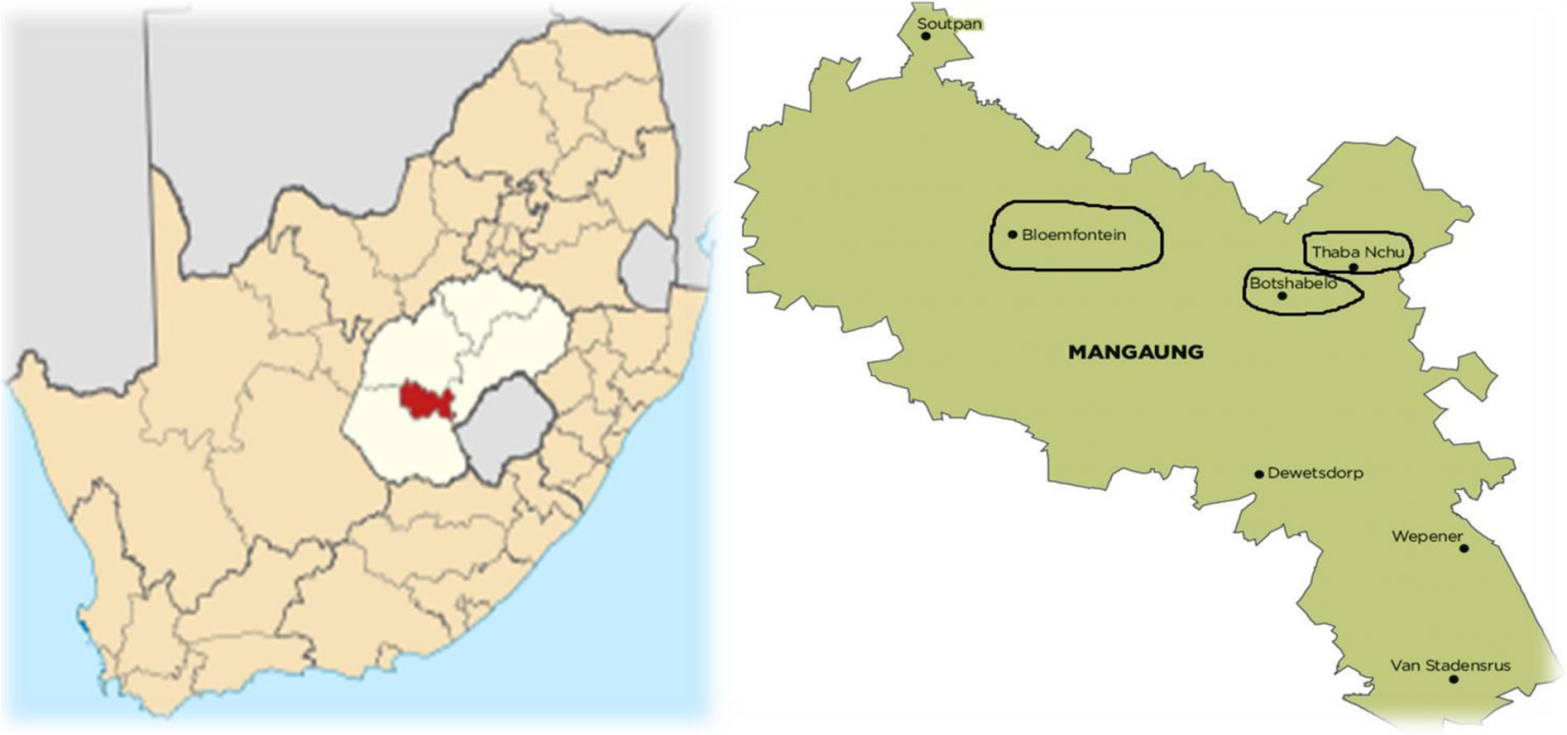
Study area (Mangaung)

A cross-sectional quantitative research design was applied in this study and permission to enter substations was obtained from Centlec, Bloemfontein. A total of 30 distribution substations (132 kV) and 30 residential sites in close proximity to chosen substations were randomly selected in three prominent areas of Mangaung Metropolitan Municipality (Bloemfontein, Botshabelo and Thaba Nchu). Out of 30 residential sites, 15 were selected in Bloemfontein (BRE1 to BRE15), nine in Botshabelo (BORE1 to BORE9) and six in Thaba Nchu (TNRE1 to TNRE6) respectively (Figs. 1, 2 and 3). Fifteen distribution substations were also selected in Bloemfontein (BS1 to BS15), nine in Botshabelo (BOS1 to BOS9) and six in Thaba Nchu (TNS1 to TNS6), and this was due to equal geographical proportion that exist in Mangaung region (Fig. 4).

The entire Mangaung region has a maximum demand of 366,800 KVA electricity, which is the highest electricity consumption in the Free State province (NERSA 2012). According to a report compiled by the South African City Networks (2014), Mangaung municipality consumes 92,710 GJ/a on buildings and facilities and a total of 142,165 GJ/a on street lightings. The electricity consumption reaches the highest peak from 4:00 am to 9:00 am in the morning. Another peak occurs from 3:00 pm to 9:00 pm in the afternoon. However, the demand and consumption usually fluctuate substantively per season (NERSA 2012). In this study, measurements were taken during summer season for duration of 20 days.

### Environmental and residential exposure sampling

**Fig. 5:**
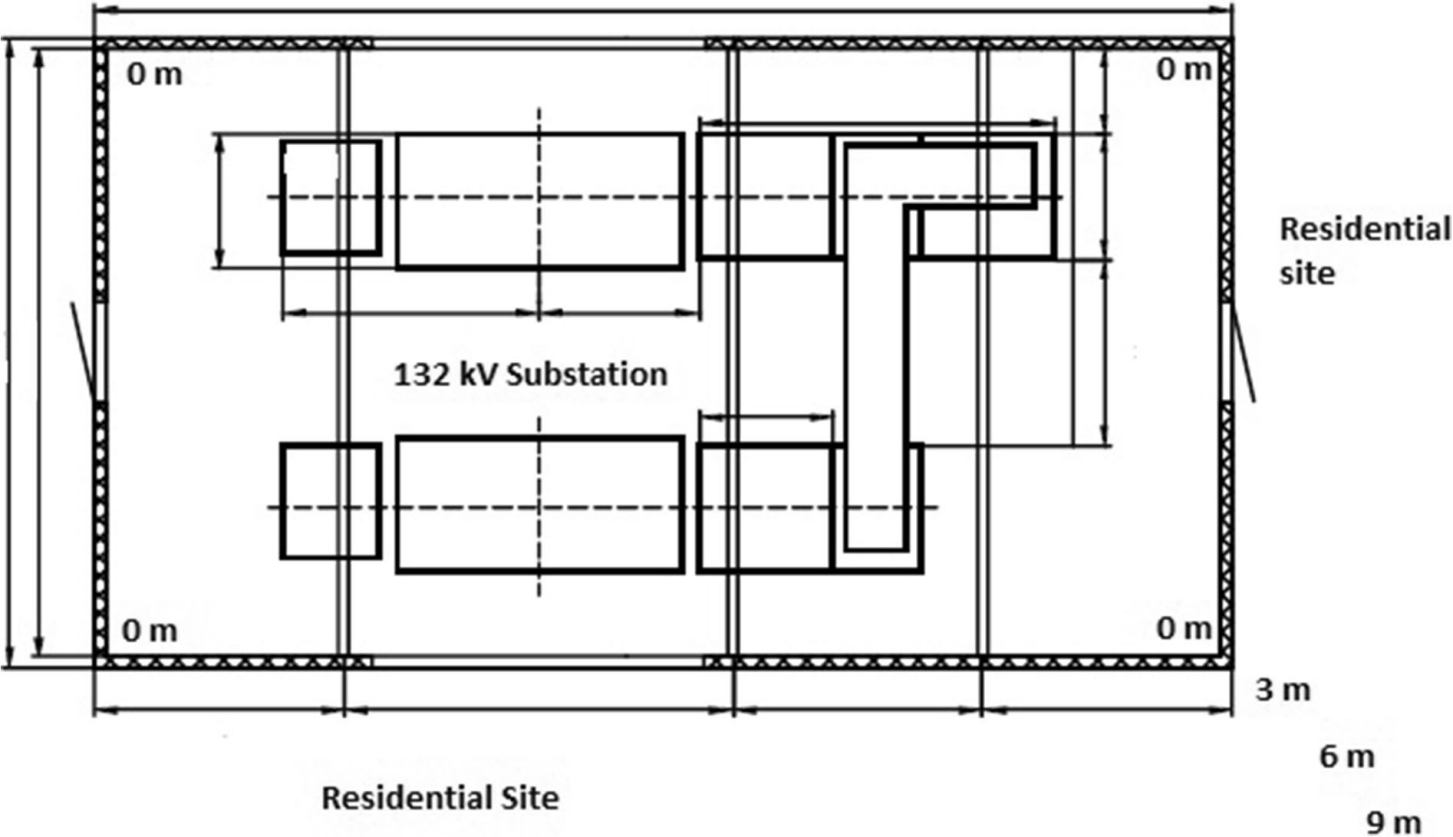
Substation and residential sampling sites

In Mangaung, substations are constructed in the residential spaces and they are located approximately 10 m from residential houses. They are enclosed with either palisade, weld mesh or pre-cast concrete fence. In this study, all four corners inside the substation, near the fence, were referred to as reference points and regarded as 0 m (see Figs. 1, 2, 3 and 5). A calibrated Trifield meter model XE 100 (frequency 3 to 3000 Hz) was used to collect measurements. A total of 120 samples were collected on distribution substations and 360 samples were collected in the residential sites on different distance interims (0 m, 3 m, 6 m and 9 m). With regard to distance interims, measurements were collected at four different corners inside each selected distribution substation, near the barrier screening, and the distance was referred to as 0 m and indicated as a reference point. Measurements were also collected at the distances of 3 m, 6 m and 9 m, near every corner outside the substations. The meter was held at a height of 1 m above the ground level to ensure that any emissions from underground power lines do not affect the readings.

At reference points (0 m) (inside the substations), the meter was held pointing to the direction of transformers. Transformers are regarded to be the main source of magnetic fields in the substations (Farag and Cheng 1994). This was also to ensure that measurements recorded are from the source of emission of electromagnetic fields (EMFs). With regard to residential sites (outside the substations), the meter was held pointing to the corners of substations for every measured distance (3 m, 6 m and 9 m) and the readings were recorded.

### Data analysis

The data obtained was recorded on the excel programme (Microsoft 2010) and further analysis were also performed using Analysis ToolPak from excel programme. Descriptive statistics was recorded for the purpose of numerical data while analytical statistics was performed to test the differences between proportions. Analysis of variance (ANOVA) and *T* test were mainly performed to compare the mean values. A significance level (*α*) of 0.05 was used.

## Results

All residential sites in Bloemfontein were compared to one another using ANOVA, including the ones in Botshabelo and Thaba Nchu to observe the significant difference in the occurrence of exposure levels. The overall occurrence of exposure levels in the residential sites of Bloemfontein was then compared to the residential sites of Botshabelo and Thaba Nchu, respectively.

Table 1 displays the descriptive statistical data of exposure levels in Bloemfontein, Botshabelo and Thaba Nchu. BORE 1, in Botshabelo, demonstrated a statistically significant difference when compared to all other residential sites (*p* < 0.001), from BORE 2 to BORE 9. BORE 4 also demonstrated the significant difference when compared to BORE 8 (*p* < 0.01) and BORE 9 (*p* < 0.006) respectively. The residential sites in Bloemfontein (BRE1 to BRE15) were compared to one another and a non-significant difference (*p* < 0.73) among all residential sites was observed. The non-significant difference (*p* < 0.35) was also observed when exposure levels in the residential sites of Thaba Nchu were compared to one another.

**Table 1:** Descriptive data and safe exposure levels statistics for ELF magnetic fields in the residential areas

Residential sites	No. of houses in close proximity	Mean (μT)	Standard deviation	Minimum	Maximum	Difference	*p* values*	Specific safe exposure levels (no health risks)
BRE 1	5	*0.42*	0.19	0.18	0.80	0.62	Non-significant difference	< 0.3 μT, no probability of leukaemia and any other form of cancer Based on exposure mean
BRE 2	3	0.28	0.11	0.12	0.48	0.36		
BRE 3	6	0.26	0.18	0.09	0.80	0.71		
BRE 4	4	0.19	0.11	0.04	0.36	0.32		
BRE 5	4	0.23	0.10	0.09	0.38	0.29		
BRE 6	5	0.16	0.12	0	0.36	0.36		
BRE 7	3	0.17	0.11	0	0.37	0.37		
BRE 8	4	0.21	0.12	0.04	0.40	0.36		
BRE 9	4	0.14	0.10	0	0.30	0.30		
BRE 10	5	0.22	0.17	0	0.50	0.50		
BRE 11	5	0.18	0.14	0	0.40	0.40		
BRE 12	4	0.21	0.11	0.02	0.39	0.37		
BRE 13	4	0.15	0.10	0	0.32	0.32		
BRE 14	4	0.15	0.12	0	0.39	0.39		
BRE 15	4	0.22	0.09	0.04	0.38	0.34		
TNRE 1	4	*0.38*	0.19	0.12	0.70	0.58	Non-significant difference	
TNRE 2	3	0.22	0.17	0.02	0.55	0.53		
TNRE 3	4	0.29	0.20	0.02	0.60	0.58		
TNRE 4	4	0.17	0.12	0	0.40	0.40		
TNRE 5	3	0.24	0.17	0	0.54	0.54		
TNRE 6	5	*0.38*	0.34	0.03	1.10	1.07		
BORE 1*	3	*0.55*	0.29	0.16	1.20	1.04	*BORE 1 to BORE9 (*p* < 0.001) *BORE 4 vs. BORE 8 (*p* < 0.01) *BORE 4 vs. BORE 9 (*p* < 0.006)	
BORE 2	3	0.26	0.53	0	1.80	1.80		
BORE 3	3	0.13	0.15	0	0.46	0.46		
BORE 4*	4	0.14	0.09	0.02	0.35	0.33		
BORE 5	5	0.15	0.10	0.02	0.36	0.34		
BORE 6	4	0.15	0.12	0	0.40	0.40		
BORE 7	4	0.17	0.13	0	0.38	0.38		
BORE 8*	4	0.22	0.10	0.08	0.42	0.34		
BORE 9*	4	0.23	0.09	0.10	0.40	0.30		

The italicized mean values indicates the probability of leukaemia (>0.3 μT)

*Significant difference was observed and *t* test was performed

The ANOVA test performed between four distance interims indicated a statistically significant difference and *T* test was performed to test the significance. Descriptive statistical data for different distance interims in Botshabelo, Bloemfontein and Thaba Nchu is indicated in Table 2.

**Table 2:** Residential magnetic field measurements at 0, 3, 6 and 9 m from the substations

Distance interims	Mean (μT)	Variance	Standard deviation	Maximum	Minimum	Difference	*p* values
0 m	0.62	0.08	0.28	1.70	0	1.70	*0 m compared to 3 m (*p* < 0.01), 6 m (*p* < 0.01) and 9 m (*p* < 0.01).
3 m	0.30	0.03	0.16	1.20	0	1.20	*3 m compared to 6 m (*p* < 0.05) and 9 m (*p* < 0.01).
6 m	0.22	0.02	0.15	0.84	0	0.84	**n/s
9 m	0.16	0.06	0.24	1.80	0	1.80	**n/s

*T test; **non-significance

A highly significant difference (*p* < 0.0001) was observed when four distance interims were compared to one another. *T* test indicated that the significant difference existed when 0 m was compared to 3 m (*p* < 0.01), 6 m (*p* < 0.01) and 9 m (*p* < 0.01), respectively. A significant difference was also observed between the exposure levels measured at 3 m and 6 m (*p* < 0.05) as well as 3 m and 9 m (*p* < 0.01). There was a non-significant difference when 6 m was compared to 9 m.

## Discussion

The exposure levels that were observed to be significantly high and at a peak were below 200 μT for general public exposures as stipulated by International Commission for Non-Ionising Radiation Protection (ICNIRP) (2010). Even though the average were below ICNIRP guidelines, exposure levels found in the residential sites of Thaba Nchu (0.28 μT) were high as compared to Botshabelo (0.22 μT) and Bloemfontein (0.21 μT) residential sites and they were all found to be non-significant as shown by the statistical tests. The high average of ELF magnetic fields found in Thaba Nchu are as a result of the peak exposure levels in TNRE 1 (0.38 μT) and TNRE 6 (0.38 μT). TNRE1 is located in proximity (10 m) to the train railway while TNRE 6 is near overhead power lines. Röösli and other researchers have demonstrated in many studies that railways emit ELF magnetic fields. Studies such as cardiovascular mortality and exposure to ELF magnetic fields: a cohort study of Swiss railway workers (Röösli et al. 2008) and leukaemia, brain tumours and exposure to ELF magnetic fields: cohort study of Swiss railway employees (Röösli et al. 2007) indicated that workers in railway industries were exposed to an average magnetic field of 120.5 μT and 21 μT. It is indicative from the said studies that the presence of railways near substations can significantly increase the exposure levels of ELF magnetic fields.

BORE 1 (0.55 μT) and BRE 1 (0.42 μT) show a high exposure levels of ELF magnetic fields and both the residential sites are located close to the 200 kV overhead power lines. A previous study by Vulevic and Osmokrovic (2011) where emission levels of magnetic fields were measured from 110, 200 and 400 kV overhead powerlines in Serbia, it was found that 110 kV power line emit 2 micro Tesla at a height of 12,6 m above the ground level and such emission increased the EMF levels in the immediate residential area of Serbia. Also, in a study conducted by Frei et al. (2013), looking at the residential distance to high-voltage power lines and risk of neurodegenerative diseases in the Danish population, it was indicated that power lines near residential environments can increase the levels of EMFs. Therefore, the above studies are indicative that the presence of power lines in the residential areas can increase the exposure levels of ELF magnetic fields. The high exposure levels observed in BORE 1, BRE 1, TNRE 1 and TNRE 6 indicate an increased probability of leukaemia to population residing in such residential sites. The WHO (2007) and IARC (2002a, b) suggest that exposure levels above 0.3 μT are associated with leukaemia cases; however, there is no conclusive evidence found.

The distance of 0 m is referred to as a reference level and it is a distance interim that exist inside the four corners of a substation, near barrier screenings. The mean exposure levels of magnetic fields were significantly higher at 0 m (0.62 μT), decreasing to 0.30 μT at 3 m, 0.22 μT at 6 m and 0.16 μT at 9 m (Table 3). Through the results obtained, this study demonstrated that ELF magnetic fields cannot be screened practically by any object in the substations and residential environments nearby. It has also been observed that the magnitude of ELF magnetic fields decreased when a distance from the source of exposure increases; however, those exposed at a distance of 0 m, which is the substation technicians, are likely to develop leukaemia. This is supported by the suggestion by WHO and IARC that exposure to ELF magnetic field levels above 0.3 μT increases the probability of leukaemia (WHO 2007; IARC 2002a, b). Huss et al. (2009) assessed mortality from neurodegenerative diseases caused by exposure to EMF from powerlines in Swiss population. Although the study looked at the health implications of exposure to EMFs from power lines, an indication that EMF exposure is relative to distance was emphasised. Among all the observations, it was noted that at a point where the overhead power line exits the substation (0 m), the exposure levels was higher (0.3 μT) than at other distance points.

**Table 3:** Exposure assessment in relation to the measured fields for one residential site

Distance intervals (from substation to residential site)	No. population exposed (number of households)	Exposure duration and time	Exposure statistics	Exposed group	Possible health effects	Study references
0 m	0	4 h- daytime; continuous emission	Range 0–1.70 μT Mean 0.62 μT SD 0.28	Occupational exposure: substation technicians	Leukaemia at exposure levels above 0.3 to 0.4 μT Based on exposure mean	WHO 2007; IARC 2002a, b
3 m	4	4 h- daytime; continuous emission	Range 0–1.20 μT Mean 0.30 μT SD 0.16	Residential exposure: households in close proximity	No probability of long-term health effects Based on exposure mean	ICNIRP 2010; IARC 2002a, b
6 m	8	4 h- daytime; continuous emission	Range 0–0.84 μT Mean 0.22 μT SD 0.15	Residential exposure: households in close proximity	No probability of long-term health effects Based on exposure mean	ICNIRP 2010; IARC 2002a, b
9 m	12	4 h- daytime; continuous emission	Range 0–1.80 μT Mean 0.16 μT SD 0.24	Residential exposure: households in close proximity	No probability of long-term health effects Based on exposure mean	ICNIRP 2010; IARC 2002a, b

## Conclusion

The results indicate that overhead power lines can significantly increase the exposure levels near substation and for future studies, source apportionment will be essential. Future studies that will investigate the health effects of exposure to ELF magnetic fields levels found in Mangaung are needed as they will provide knowledge on exposure and health effects among residential households. As the substations are near residential households in Mangaung, EMFs awareness campaigns are needed to alert the general public about the influence of duration and frequency of exposure in the development of health effects. In this study, the measurements were collected during summer season and the probability of exposure levels to increase in other seasons such as winter is significantly high. This is due to increased use of electricity during winter resulting in high consumption of electricity and substantial fluctuation.

Increased distance has played a significant role in the reduction of magnitude of ELF magnetic fields. There is a need for the development of a model that will address the short-term exposure limits in relation to EMFs and also 200 μT limit of ICNIRP to be measured against time. It has been confirmed in many studies that the evidence with regard to association between exposure to ELF magnetic fields and development of health impact is not conclusive. However, there is a need in South Africa to develop a legislation that reduces residential exposure to ELF magnetic fields from either substations or power lines. Such legislation should stipulate specific safe exposure limits informed by epidemiological studies. Currently, exposure to EMFs in South Africa has been reduced through the use of ICNIRP guidelines.

### Significance

The results from this study are preliminary and noteworthy. Although the exposure levels are below ICNIRP guidelines, but this study creates public awareness on the exposure to ELF magnetic fields in the residential environments of Mangaung metropolitan municipality. The data obtained is also significant in the field of environmental health as it forms the basis of health promotion around exposure to non-ionising radiation.
